# Comparative Effects of Monascin and Monascinol Produced by *Monascus pilosus* SWM-008 on Pro-Inflammatory Factors and Histopathological Alterations in Liver and Kidney Tissues in a Streptozotocin–Nicotinamide-Induced Rat Model

**DOI:** 10.3390/jof10120815

**Published:** 2024-11-25

**Authors:** Pei-Xin Yang, Ya-Wen Hsu, Tzu-Ming Pan, Chun-Lin Lee

**Affiliations:** 1 Department of Life Science, National Taitung University, Taitung 95092, Taiwan; px.yang@sunway.cc; 2 SunWay Biotech Co., Taipei 11494, Taiwan; 3 Department of Biochemical Science and Technology, National Taiwan University, Taipei 10617, Taiwan

**Keywords:** liver and kidney damage, *Monascus pilosus*, monascinol, monascin, streptozotocin and nicotinamide

## Abstract

Monascinol (Msol), an analog of monascin (MS) produced by *Monascus pilosus*, possesses potential anti-inflammatory properties. This study compares the effects of *M. pilosus* SWM-008 fermented red mold rice, which contains the functional components MS and Msol, on liver and kidney damage related to diabetic complications in rats. An animal model of liver and kidney injury was induced by an intraperitoneal injection of streptozotocin (STZ) at 65 mg/kg body weight combined with nicotinamide (NA) at 150 mg/kg body weight. Our findings indicate that Msol significantly reduces STZ-NA induced pro-inflammatory markers, including interleukin-1β (IL-1β), interleukin-6 (IL-6), tumor necrosis factor-α (TNF-α), and cyclooxygenase-2 (COX-2) in both liver and kidney tissues. Significant improvements were noted in the histopathological assessments. Msol was more effective than MS in suppressing renal IL-1β and COX-2 expressions. In summary, the findings indicate that Msol shows potential as a novel therapeutic agent for treating liver and kidney injuries associated with diabetic complications.

## 1. Introduction

The characteristic of diabetes is the elevation of blood glucose levels and the occurrence of complications. One common complication is diabetic liver disease (DLD), which refers to the damage to liver tissue and impaired liver function caused by diabetes, including non-alcoholic fatty liver disease, cirrhosis, and progression to hepatocellular carcinoma [1]. The liver is an important site for the metabolism of carbohydrates, lipids, and proteins in the animal body. The symptoms of hyperglycemia produced by diabetes, oxidative stress caused by high blood glucose, and inflammatory responses induced by hyperglycemia are all factors that contribute to liver damage [2]. The liver produces approximately 90% of endogenous glucose in the human body through hepatic glucose production (HGP) [3]. After a meal, high blood glucose levels trigger glycogen synthesis and storage in the liver, while during low blood glucose, gluconeogenesis and glycogenolysis are initiated to provide sufficient glucose energy [4,5]. In addition, hepatic glucose regulation is also influenced by insulin and glucagon, and insulin secretion promotes the storage of lipids and carbohydrates. In summary, diabetic patients may develop liver disease due to the accumulation of excess glucose in the liver caused by high blood glucose. In addition, diabetic kidney disease (DKD) is also a common complication of diabetes. Diabetic patients have a much higher risk of developing kidney disease compared to non-diabetic individuals. The kidneys are organs that are susceptible to damage caused by high blood glucose levels. Prolonged high blood glucose levels can increase oxidative stress in the body, leading to the production of reactive oxygen species (ROS) [6]. When the kidneys are under stress, it can cause glomerular expansion, sclerosis, interstitial fibrosis, and inflammation. These symptoms are common in end-stage renal disease, making diabetic nephropathy (DN) one of the most serious complications of diabetes [7,8].

Red mold rice (RMR) is a traditional *Monascus*-fermented food, and nowadays the industrial fermentation of RMR is mostly carried out by three species: *M. purpureus*, *M. ruber*, and *M. pilosus* [9]. Numerous studies have confirmed the health benefits of *Monascus*. For instance, in cell experiments conducted by Liu et al., RMR extracts were found to reduce cellular aging induced by high glucose, primarily by increasing the activity of intracellular antioxidant enzymes glutathione (GSH) and glutathione reductase (GRd), which help reduce intracellular ROS. Additionally, it was found to enhance the expression of heme oxygenase-1 (HO-1) and thereby reduce oxidative stress caused by high glucose [10]. The study by Rajasekaran also showed that *M. purpureus*-fermented RMR can improve oxidative stress and hyperlipidemia in the kidneys of streptozotocin (STZ)-induced rats, thereby slowing down diabetic complications [11]. In addition, RMR fermentation products contain various functional ingredients during the fermentation process, including deferricoprogen (DFC), dimerumic acid (DMA), monascin (MS), ankaflavin (AK), monacolin K (MK), monascinol (Msol), and γ-amino butyric acid (GABA) [9,12,13,14]. MS and AK are the main yellow pigments produced by RMR. MS and AK have been shown to have antioxidant [15,16] and anti-inflammatory effects [17,18]. And Msol, also known as monascuspiloin, is a yellow pigment similar to MS [19]. MS and Msol are azaphilonoid pigments produced by *M. pilosus* during the fermentation of red mold rice. MS has a molecular formula of C_21_H_26_O_5_ and a molecular weight of approximately 358.43 g/mol, while Msol has a molecular formula of C_21_H_28_O_5_ and a molecular weight of about 360.44 g/mol. Both compounds exhibit good solubility in organic solvents such as ethanol and methanol but have limited solubility in water, which may affect their bioavailability. They are relatively stable under acidic conditions but may degrade under alkaline conditions [17,18,19]. Exposure to light and high temperatures can lead to structural decomposition, impacting their stability. According to previous studies, Msol has the potential to treat prostate cancer [19,20] and can regulate gene transcription and protein expression related to lipid regulation and oxidative stress in the liver, treating alcohol-induced liver injury in mice [21]. However, there is currently no literature available on the effects of Msol in improving liver and kidney damage caused by STZ combined with nicotinamide (NA).

MS has been previously validated for its blood glucose regulation capabilities alongside significant antioxidative and anti-inflammatory effects. However, this study uncovers another component within RMR, Msol, which may exhibit similar functionalities. Given its chemical structure, Msol is posited to possess superior antioxidative properties. Consequently, this research aims to investigate the less explored novel yellow pigment, Msol, for its potential in ameliorating oxidative damage and inflammatory responses in the liver and kidneys induced by STZ-NA. This study signifies an important step towards unveiling a new functional ingredient in *Monascus* fermentation products, potentially offering new therapeutic strategies and opportunities for health product development in related fields.

## 2. Materials and Methods

### 2.1. Chemicals

Msol and MS (99.9% purity) were provided by Sunway Biotechnology Co (Taipei, Taiwan, ROC) and were extracted and purified from *Monascus pilosus* SWM-008-fermented RMR. STZ and NA were purchased from Sigma Chemical Co. (St. Louis, MO, USA). Sodium citrate was purchased from Shimakyu’s Pure Chemicals (Osaka, Japan). Tumor necrosis factor-α (TNF-α) antibody (80045-T50), TNF-α standard protein (80045-RNAE), interleukin-6 (IL-6) antibody (80076-T16), IL-6 standard protein (50136-MNAE), interleukin-1β (IL-1β) antibody (80023-RP02), and IL-1β standard protein (50101-MNAE) were purchased from Sino Biological Inc. (Beijing, China). Cyclooxygenase-2 (COX-2) antibody (12375-1-AP), COX-2 standard protein (Ag24721), and HRP-conjugated affinipure goat anti-rabbit IgG (H + L) (SA00001-2) were purchased from Proteintech (Chicago, IL, USA).

### 2.2. Animal Grouping and Experiment Schedule

Sixty-four male Sprague Dawley (SD) rats, aged 6 weeks, were purchased from BioLasco Co. (Taipei, Taiwan). The rats were housed in individual plastics cages, placed in a room on a 12 h light/dark cycle, and maintained at a temperature of 25 °C and 60% relative humidity. The animals were reared for one week with free access to food and water to adapt to the new environment. After one week, the rats were induced with STZ + NA by injection. The doses of STZ combined with NA in this experiment were adjusted based on Chen’s study and previous experimental records from our laboratory [22]. The animals were fasted for 10–12 h prior to injection and then given the protective agent NA (150 mg/kg b.w.) via intraperitoneal injection. After resting for 15–30 min, STZ (65 mg/kg b.w.) was administered. NA was dissolved in physiological saline solution before injection, and STZ was prepared 10 min prior to injection by dissolving it in a physiological saline solution containing 10 mM sodium citrate (pH 4.2–4.5) to prevent degradation. The experimental substances in *Monascus*-fermented RMR, Msol and MS, were analyzed by previous studies conducted in our laboratory. The functional MS component content in the RMR was found to be 6 mg/g, while the Msol content was 3 mg/g. Based on the experimental results, an animal dosing design was established, equivalent to an adult daily intake of 0.5–2 g of RMR [23].

After induction, the rats were randomly divided into eight groups based on their fasting blood glucose levels: normal non-induced (NOR group), STZ-NA injection group (STZ-NA group), STZ-NA + low dose of RMR (RL group) treated with 51.67 mg/kg b.w./rat/day of RMR equivalent to 0.5 g/day for a 60 kg adult, STZ-NA + high dose of RMR (RH group) treated with 206.67 mg/kg b.w./rat/day of RMR equivalent to 2 g/day for a 60 kg adult, STZ-NA + 1-fold dose Msol (Msol-1X group) treated with 0.31 mg/kg b.w./rat/day of Msol equivalent to 3 mg/day for a 60 kg adult, STZ-NA + 2-fold dose Msol (Msol-2X group) treated with 0.62 mg/kg b.w./rat/day of Msol equivalent to 6 mg/day for a 60 kg adult, STZ-NA + 1-fold dose MS (MS-1X group) treated with 0.31 mg/kg b.w./rat/day of MS equivalent to 3 mg/day for a 60 kg adult, STZ-NA + 2-fold dose MS (MS-2X group) treated with 0.62 mg/kg b.w./rat/day of MS equivalent to 6 mg/day for a 60 kg adult by oral gavage, respectively. Meanwhile, NOR and STZ-NA groups were both daily given ultra-pure water by oral gavage. The experiment lasted for 8 weeks. During the experiment, animal body weight, water, and food intake were recorded weekly. This animal experiment was reviewed and approved by the Institutional Animal Care and Use Committee (IACUC) of the National Taitung University (approved no. NTTU 110007).

**Figure 1:**
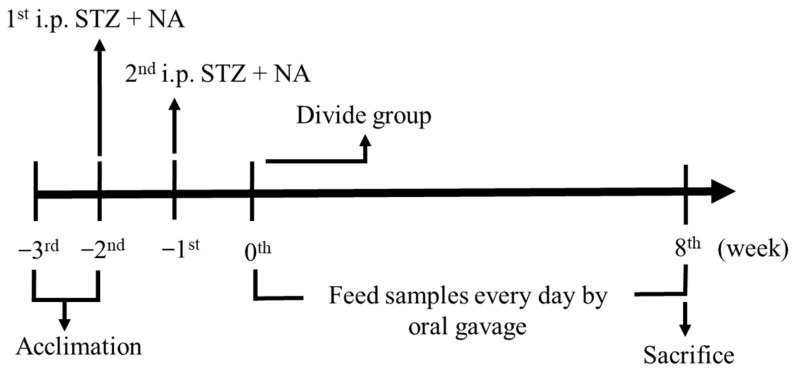
The schedule of STZ + NA diabetic rat induction.

After 8 weeks, the rats were fasted for 12 h and sacrificed by CO_2_ asphyxiation. After blood withdrawal from the posterior vena cava, the blood was placed into eppendorfs, stored at 25 °C for 2 h, and centrifuged at 10,000× *g* for 10 min at 4 °C to obtain the serum. The samples were then stored at 4 °C and sent for analysis the next day by Accuspeedy Medical Laboratory (Tainan, Taiwan). Liver tissue and kidney tissue were collected, some of which was kept in 10% formalin for pathology to facilitate subsequent section staining, and some of which was rinsed with 0.9% normal saline to remove excess blood. The liver and kidney tissues were stored at −80 °C for further analysis. During the experiment, animal body weight and water and food intake were recorded weekly. The experimental schedule is shown in Figure 1.

### 2.3. Serum Liver Function Index (AST, ALT, Albumin) and Renal Function Index (BUN, Creatinine, Uric Acid) Concentration

The serum liver function index (aspartate aminotransferase (AST), alanine trans-aminase (ALT), and albumin) and renal function index (blood urea nitrogen (BUN), creatinine, uric acid) were entrusted to Accuspeedy Medical Laboratory (Tainan, Taiwan), which measured using a chemistry analyzer (c702, Roche, Basel, Switzerland) and the following kits were used: AST assay kit (0005850819190c701, cobas^®^, Basel, Switzerland), ALT assay kit (0005850797190c701, cobas^®^, Basel, Switzerland), albumin assay kit (0005166861190c701, cobas^®^, Basel, Switzerland), BUN assay kit (0105171873190c701, cobas^®^, Basel, Switzerland), creatinine assay kit (0006407137190c701, cobas^®^, Basel, Switzerland), uric acid assay kit (0005171857190c701, cobas^®^, Basel, Switzerland). The method is as shown in the instructions.

### 2.4. H&E Staining

After soaking the second largest lobe of the liver and the kidney in 10% formalin fixation, tissue sections were prepared by slicing, followed by washing, dehydration, and embedding in paraffin. The tissue sections were then stained using hematoxylin and eosin (H&E) staining.

### 2.5. Determination of Liver and Kidney Protein Expression

#### 2.5.1. Tissue Homogenization

Liver or kidney tissue (0.1 g) was weighed and homogenized with 1 mL of PBS (0.026 M NaCl, 0.0026 M NaH_2_PO_4_, pH 7). The homogenate was centrifuged at 12,000× *g* for 15 min at 4 °C and the supernatant was used for further analysis. Total protein in lysates was determined using the BCA Protein Assay Kit (71285-3, Merck, Kenilworth, NJ, USA) according to the manufacturer’s instructions.

#### 2.5.2. Target Protein (TNF-α, IL-6, IL-1β, and COX-2) Analysis

The target protein analysis was performed using enzyme-linked immunosorbent assay (ELISA) for the following proteins: TNF-α, IL-6, IL-1β, and COX-2. The experimental procedure was modified based on the previous study [24]. The analysis procedure was as follows: 100 μL of sample homogenate or different concentrations of protein standards were added to a 96-well plate and incubated at 37 °C for 1 h. The samples or protein standards were then removed. After adding 100 μL of primary antibody, the plate was incubated at 37 °C for 1 h. One hour later, the primary antibody was removed, and the plate was washed three times with PBST (500 mL PBS, 0.05% Tween 20). The plate was inverted on a paper towel and gently tapped to completely remove the residual liquid. Subsequently, 100 μL of secondary antibody was added, and the plate was incubated at 37 °C for 1 h. After one hour, the antibody was removed, and the plate was washed six times with PBST, inverted on a paper towel, and gently tapped to remove any remaining liquid. Finally, 100 μL of the substrate (3,3′,5,5′-tetramethylbenzidine, TMB) was added and incubated at 37 °C for 15 min. The reaction was stopped by adding 50 μL of 2N sulfuric acid, and the absorbance was measured at a 450 nm wavelength.

The protein expression levels of the target proteins in the samples were determined by fitting the absorbance values into the standard curve generated using the protein standards.

### 2.6. Statistical Analysis

Data are expressed as means ± standard deviation. The statistical one-way analysis of variance (ANOVA) was determined by using SPSS version 12.0 software (SPSS Institute, Inc., Chicago, IL, USA) with Duncan’s multiple range test. Differences with *p* < 0.05 were considered statistically significant.

## 3. Results

### 3.1. Effects of SWM-008 Red Mold Rice and Functional Components on Changes in Body Weight, Liver Weight, Kidney Weight, Food Intake, and Water Consumption in STZ-NA-Induced Rats

This study used STZ combined with NA to induce liver and kidney damage in the rat model. Table 1 shows the initial body weight, final body weight, liver weight, and kidney weight of the rats at the beginning of the experiment. There were no significant differences in initial body weight among the groups before STZ-NA induction (*p* < 0.05). In the end, there was a decrease in liver weight and body weight (*p* < 0.05). Table 2 shows the total food intake and total water intake during the experiment. It was observed that STZ-NA-induced rats exhibited symptoms of increased food and water intake and decreased body weight. There were no significant differences (*p* > 0.05) in total food intake, water intake, liver weight, and final body weight between the STZ-NA group and the various treatment groups.

### 3.2. Effects of SWM-008 Red Mold Rice and Functional Components on Serum Liver Function Indicators in STZ-NA-Induced Rats

Table 3 displays liver indicators in the rat serum, including AST and ALT, which are enzymes produced by the liver. The results show that in the STZ-NA group of rats, the levels of AST and ALT in the body are significantly higher than those in the NOR group (*p* < 0.05). However, feeding SWM-008 RMR exhibits a dose-dependent reduction in their levels, and the pure substances Msol and MS also decrease the serum activity of AST and ALT (*p* < 0.05). In the STZ-NA group, the albumin levels are significantly lower compared to the NOR group (*p* < 0.05). However, in the RMR-fed groups, the albumin levels in the serum can be restored in a dose-dependent manner (*p* < 0.05), with a slight trend of increase observed in the Msol-1X and Msol-2X groups. The MS-1X and MS-2X groups exhibit significant differences compared to the STZ-NA group (*p* < 0.05). These findings demonstrate that RMR has the ability to inhibit the elevation of liver damage markers associated with diabetes complications and protect the liver from damage. The active compounds Msol and MS both show improvements in these symptoms, with MS exhibiting superior restorative effects.

### 3.3. Effects of SWM-008 Red Mold Rice and Functional Components on the Pro-Inflammatory Factors IL-6, IL-1β, TNF-α, and COX-2 in the Liver of STZ-NA-Induced Rats

**Figure 2:**
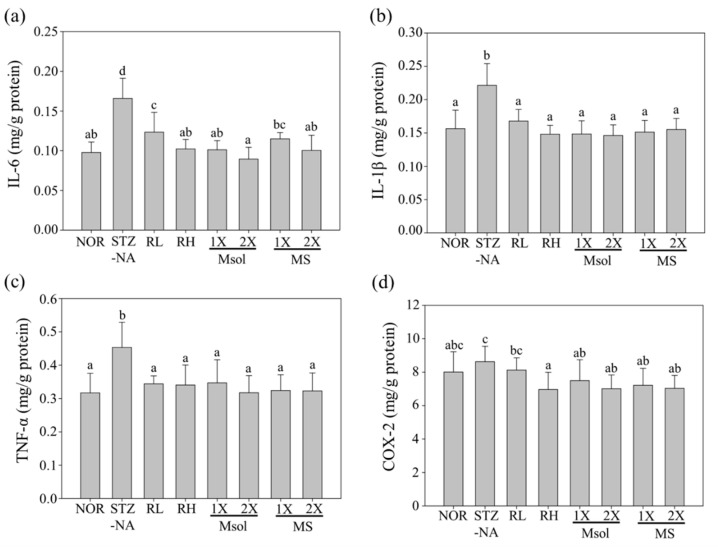
Effect of different dosages of *Monascus pilosus* SWM-008-fermented red mold rice, monascinol, and monascin on the levels of pro-inflammatory (**a**) IL-6, (**b**) IL-1β, (**c**) TNF-α, and (**d**) COX-2 expressions on the liver in STZ-NA-induced rats. Two groups of the rats daily administered with RO water (NOR group) or i.p. injected with 65 mg/kg b.w. STZ + 150 mg/kg b.w. NA to induce liver and kidney damage and daily administered with RO water (STZ-NA group). The other STZ-NA-induced rats were administered with a low dose (51.67 mg/kg b.w./day) (RL group) or high dose of *Monascus pilosus* SWM-008-fermented red mold rice (206.67 mg/kg b.w./day) (RH group), 1-fold dose monascinol (0.31 mg/kg b.w./day) (Msol-1X group), 2-fold dose monascinol (0.62 mg/kg b.w./day) (Msol-2X group), 1-fold dose monascin (0.31 mg/kg b.w./day) (MS-1X group), and 2-fold dose monascin (0.62 mg/kg b.w./day) (MS-2X group). Data are presented as means ± SD (n = 8). Mean values with different letters are significantly different (*p* < 0.05). IL-6: interleukin-6, IL-1β: interleukin-1β, TNF-α: tumor necrosis factor-α, COX-2: cyclooxygenase-2.

Figure 2 illustrates the impact of RMR and its functional components on the hepatic expression levels of pro-inflammatory factors IL-6, IL-1β, TNF-α, and COX-2 in STZ-NA-induced rats. Compared to the NOR group, the hepatic IL-6 content significantly increased by 70% in the STZ-NA-induced rats (*p* < 0.05). All experimental groups receiving the test substances exhibited a significant reduction in hepatic IL-6 activity (*p* < 0.05), displaying a dose-dependent effect. RL and RH levels decreased by 26% and 33%, respectively. Msol at 1X and 2X doses reduced levels by 39% and 46%, while MS at 1X and 2X doses reduced them by 31% and 40%. Among these, the double dose of Msol showed the most significant effect (Figure 2a). The results of IL-1β and TNF-α are shown in Figure 2b,c. Compared with NOR, the STZ-NA group significantly increased the activities of IL-1β and TNF-α by 42% and 43%, respectively. Each group fed the test substance effectively reduced IL-1β and TNF-α expression in the liver (*p* < 0.05), with no significant difference among the groups.

Figure 2d shows the expression levels of COX-2 in the liver. In STZ-NA -induced rats, the COX-2 content only showed a slight increase compared to the NOR group. However, the test substances, when compared to the STZ-NA group, significantly reduced COX-2 expression at double the dosage of RMR and with both Msol and MS (*p* < 0.05). These results confirm that STZ-NA induction leads to a significant increase in liver protein expression of IL-6, IL-1β, and TNF-α, indicating an inflammatory response. However, both RMR and its active components, Msol and MS, were effective in reducing the expression levels of these pro-inflammatory factors, achieving an anti-inflammatory effect.

### 3.4. Effects of SWM-008 Red Mold Rice and Functional Components on Liver Histopathology in STZ-NA-Induced Rats

**Figure 3:**
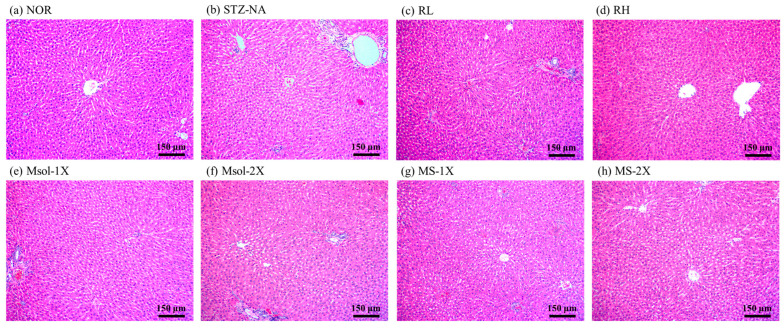
Effects of different dosages of *Monascus pilosus* SWM-008-fermented red mold rice, monascinol, and monascin on liver pathological changes in the STZ-NA-induced rats. Representative images of H&E-stained sections (original magnifications X100). (**a**) NOR, (**b**) STZ-NA, (**c**) RL, (**d**) RH, (**e**) Msol-1X, (**f**) Msol-2X, (**g**) MS-1X, (**h**) MS-2X.

The results of H&E pathological staining of liver tissue at 100× magnification are shown in Figure 3. In this experiment, although the serum data and liver inflammation protein data indicated liver damage in STZ-NA-induced rats, the liver did not exhibit significant pathological changes. Furthermore, there were no pathological alterations observed in any of the test substance groups.

### 3.5. Effects of SWM-008 Red Mold Rice and Functional Components on Serum Kidney Function Indicators in STZ-NA-Induced Rats

The serum BUN concentration, as shown in Table 4, indicates that the BUN levels in the STZ-NA group are significantly higher than those in the NOR group (*p* < 0.05). This confirms that STZ-NA induction leads to substantial kidney damage. Feeding the test substances, both low and high doses of RMR, effectively reduced the BUN levels (*p* < 0.05). However, it was necessary to administer Msol at a double dose to observe a decreasing trend. Table 4 also demonstrates that creatinine in each group after STZ-NA induction was significantly reduced compared with the NOR group (*p* < 0.05). After STZ-NA induction, the STZ-NA group exhibited significantly lower uric acid levels compared to the NOR group (*p* < 0.05). Feeding RMR did not significantly improve uric acid levels, but both the Msol and MS groups showed a significant increase in uric acid levels (*p* < 0.05). In summary, based on the above results, RMR and its active components, Msol and MS, all have protective effects against kidney damage induced by STZ-NA.

### 3.6. Effects of SWM-008 Red Mold Rice and Functional Components on the Pro-Inflammatory Factors IL-6, IL-1β, TNF-α, and COX-2 in the Kidney of STZ-NA-Induced Rats

Diabetes can cause severe kidney disease, and in the early stages of the disease, it can lead to kidney inflammation. As indicated by the serum renal function indicators in Table 4, this experiment demonstrates that STZ-NA induction has resulted in kidney damage and complications. Therefore, we measured kidney inflammation-related proteins such as IL-6, IL-1β, TNF-α, and COX-2 expression levels to evaluate whether renal inflammation occurred and to assess the effect of RMR and its functional ingredients on improving renal inflammation.

**Figure 4:**
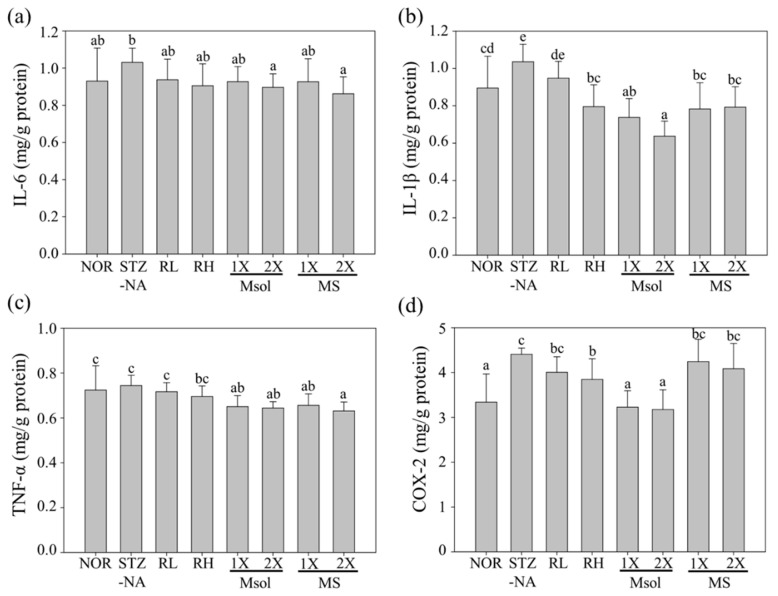
Effect of different dosages of *Monascus pilosus* SWM-008-fermented red mold rice, monascinol, and monascin on the levels of pro-inflammatory (**a**) IL-6, (**b**) IL-1β, (**c**) TNF-α, and (**d**) COX-2 expressions on the kidney in STZ-NA-induced rats. Two groups of the rats daily administered with RO water (NOR group) or i.p. injected with 65 mg/kg b.w. STZ + 150 mg/kg b.w. NA to induce liver and kidney damage and daily administered with RO water (STZ-NA group). The other STZ-NA-induced rats were administered with a low dose (51.67 mg/kg b.w./day) (RL group) or high dose of *Monascus pilosus* SWM-008-fermented red mold rice (206.67 mg/kg b.w./day) (RH group), 1-fold dose monascinol (0.31 mg/kg b.w./day) (Msol-1X group), 2-fold dose monascinol (0.62 mg/kg b.w./day) (Msol-2X group), 1-fold dose monascin (0.31 mg/kg b.w./day) (MS-1X group), and 2-fold dose monascin (0.62 mg/kg b.w./day) (MS-2X group). Data are presented as means ± SD (n = 8). Mean values with different letters are significantly different (*p* < 0.05). IL-6: interleukin-6, IL-1β: interleukin-1β, TNF-α: tumor necrosis factor-α, COX-2: cyclooxygenase-2.

The results of the impact of SWM-008 RMR and its active components on the kidney pro-inflammatory factors IL-6, IL-1β, TNF-α, and COX-2 in STZ-NA-induced rats are shown in Figure 4. Compared to the NOR group, the STZ-NA group exhibited a slight increase of 11% in kidney IL-6 expression and a significant increase in IL-1β and COX-2 levels, which rose by 16% and 26%, respectively (*p* < 0.05). Feeding RMR can induce a dose-dependent reduction in the expression of IL-6, IL-1β, and COX-2 (*p* < 0.05). Pure Msol and MS both reduced IL-6 expression to a level that was not significantly different from the NOR group (*p* > 0.05). However, in terms of IL-1β and COX-2 expression at the same dosage, Msol had a more favorable effect compared to the MS group. In this experiment, STZ-NA induction did not increase the expression of TNF-α in the kidneys (*p* > 0.05). However, feeding RMR at a high dosage could slightly reduce TNF-α expression, and pure Msol and MS at a one-fold dosage significantly reduced the expression of TNF-α (*p* < 0.05).

Based on the above results, it can be concluded that STZ-NA induction does not cause as significant an inflammatory response in the kidneys as it does in the liver. However, there is still a slight elevation in the expression levels of the pro-inflammatory factors IL-6, IL-1β, TNF-α, and COX-2. Feeding RMR at a dosage of 2 times the regular amount is required to significantly reduce the inflammatory factors elevated by STZ-NA induction. Among the substances tested, Msol demonstrated the most significant improvement, suggesting that Msol is the primary active substance in RMR for reducing kidney inflammatory factors.

### 3.7. Effects of SWM-008 Red Mold Rice and Functional Components on Kidney Histopathology in STZ-NA-Induced Rats

**Figure 5:**
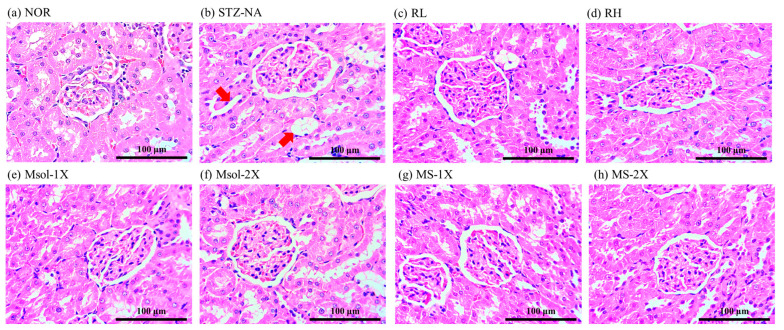
Effects of different dosage of *Monascus pilosus* SWM-008-fermented red mold rice, monascinol, and monascin on kidney pathological changes in the STZ-NA-induced rats. Representative images of H&E-stained sections (original magnifications X400). (**a**) NOR, (**b**) STZ-NA, (**c**) RL, (**d**) RH, (**e**) Msol-1X, (**f**) Msol-2X, (**g**) MS-1X, and (**h**) MS-2X. The red arrow is STZ-NA-induced minimal to moderate hydropic degeneration of the kidney tubules.

The H&E pathological staining results of kidney tissue at a 400× magnification are shown in Figure 5. In the NOR group, there were no apparent signs of damage or abnormalities in the kidney. After STZ-NA induction, the STZ-NA group showed partial effects on kidney glomeruli with no significant impact, but the renal tubules exhibited swelling and expansion (indicated by the red arrows), resulting in irregular distribution of kidney cells due to compression. Feeding RMR showed a noticeable improvement in renal tubule swelling, with the most significant improvement observed at 2 times the regular RMR dosage. Pure Msol and MS also showed a slight improvement in the condition.

## 4. Discussion

Currently, there are various induction methods for animal models of diabetes, with the chemical agent STZ being a common method used to disrupt the pancreatic β cells in experimental animals, leading to a decrease in insulin secretion [25]. Administering a single high dose or multiple low doses of STZ to induce symptoms in experimental animals can mimic the characteristics of human type 1 diabetes [26]. However, when co-administered with NA, STZ’s toxic effects on pancreatic β cells can be mitigated, preserving a certain number of β cell activities and resulting in insulin secretion impairment characteristic of diabetes [27,28]. Furthermore, the serum analysis results from this study also demonstrate that STZ-NA induction can lead to complications in liver and kidney functions. Taken together, these findings indicate that STZ-NA induction can successfully induce liver and kidney damage in diabetic animals

Long-term high blood glucose can lead to an increase in oxidative stress within the body, resulting in chronic inflammation. Injection of STZ will induce nuclear factor kappa-light-chain-enhancer of activated B cells (NF-κB) activation, subsequently affecting the increase in pro-inflammatory factors such as IL-6, IL-1β, TNF-α, and COX-2, leading to oxidative stress and inflammatory responses, resulting in cellular damage [25]. These inflammatory factors can lead to the degradation of insulin receptor substrate 1 (IRS1) in the liver, reducing insulin sensitivity, ultimately leading to the development of insulin resistance and elevated blood glucose levels [29,30]. Liver enzymes such as AST and ALT are released into the bloodstream when liver damage occurs, resulting in elevated levels [31,32]. Albumin, produced in the liver, decreases in serum levels when liver cells are damaged due to reduced production. Conditions like liver and kidney disease, as well as nutritional issues, can lead to low albumin levels [33,34]. In STZ-NA-induced rat in this experiment, abnormal hepatic glycogen accumulation and chronic hyperglycemia led to elevated liver enzyme activities of AST and ALT, as well as a significant decrease in albumin content (Table 3). Additionally, there was a notable increase in hepatic inflammatory factors (IL-6, IL-1β, TNF-α, and COX-2) in the STZ-NA group compared to the NOR group, confirming that STZ-NA induced liver inflammation responses resulting in liver damage (Figure 2). Administration of high-dose RMR improved AST, ALT, and albumin levels most significantly. Furthermore, RMR was also effective in reducing the expression of hepatic inflammatory factors such as IL-6, IL-1β, TNF-α, and COX-2. The pure Msol significantly reduced AST and ALT levels, regardless of whether it was administered at a 1X or 2X dosage, but it did not show a dose–response effect. Msol also demonstrated a significant reduction in the expression of inflammatory factors induced by STZ and NA. In the Msol-2X group, the reduction in IL-6 expression was most pronounced, even slightly lower than that of the NOR group.

Diabetic complications not only cause damage to the liver, but the symptoms of hyperglycemia can easily lead to kidney complications, including glomerular expansion, sclerosis, and interstitial fibrosis inflammation in the kidneys [7,8]. Therefore, common kidney function indicators such as BUN, creatinine, and uric acid are measured to assess the condition of the kidneys. BUN represents metabolic waste products from protein utilization within cells, while creatinine is primarily a metabolic byproduct produced during muscle activity. Both of these substances need to be filtered by the kidneys and excreted in urine. When both values increase, it indicates an abnormality in kidney function [35]. In Table 4, we observed abnormal values in kidney function indicators in the STZ-NA group, including an increase in BUN and a decrease in creatinine and uric acid levels. The elevated BUN levels indeed suggest that the symptoms of hyperglycemia in diabetes have contributed to this increase. The decrease in creatinine levels is likely due to significant weight loss induced by diabetes, resulting in reduced muscle mass and subsequently lowering serum creatinine levels. The decrease in uric acid levels aligns with some literature indicating that uric acid levels tend to rise in the early stages of diabetes but decline as the disease progresses [36]. This suggests that the diabetes experiment has reached an advanced stage, and the overall values indicate that kidney function has been affected by diabetes, resulting in some kidney damage. After feeding the rats with SWM-008-fermented RMR, there was a decrease in BUN levels, and uric acid levels showed slight improvement. When administering pure Msol, it only led to a slight improvement in BUN levels but significantly restored uric acid levels. MS, on the other hand, outperformed Msol in improving BUN levels and also significantly restored uric acid levels. However, none of the test substances in any group were able to improve creatinine levels. This is likely because the overall body weight data did not differ from the STZ-NA group, and there was no weight recovery observed.

The serum data confirmed the occurrence of kidney damage, and histopathological examination also revealed multiple instances of tubular swelling and expansion in the kidneys of STZ-NA-induced rats in this experiment. Conditions such as tubular dilation and glomerular enlargement are well-established as typical markers of diabetic kidney injury in research studies [37,38]. Feeding the experimental subjects with the test substances showed a noticeable improvement in these effects, with the results at a high dosage of RMR approaching those of the NOR group in the histological slices. Furthermore, previous studies have identified that the primary cause of diabetic nephropathy is the long-term elevation of intracellular glucose levels due to high blood sugar. This elevation prompts the self-oxidation of glucose, leading to the production of advanced glycation end products (AGEs), resulting in an increase in ROS. This induces the substantial expression of pro-inflammatory factors TNF-α, IL-1β, and IL-6 in the kidneys [39]. In addition, several studies have confirmed that the yellow pigment MS possesses significant benefits, including the ability to improve hyperglycemia, reduce insulin resistance, and provide anti-inflammatory and antioxidant effects [40,41,42]. In our study, STZ-NA indeed led to an increase in the expression levels of IL-1β, IL-6, and COX-2 in the kidneys of the STZ-NA group. However, feeding rats with SWM-008-fermented RMR effectively reduced the expression of IL-1β, IL-6, and COX-2. Moreover, the pure Msol significantly decreased the expression of renal inflammatory factors TNF-α, IL-1β, IL-6, and COX-2. Notably, at the same dosage, Msol exhibited a more pronounced improvement in IL-1β and COX-2 expression compared to MS. These findings confirm that SWM-008 RMR, along with its functional components Msol and MS, can effectively inhibit the expression of inflammatory factors, achieving a therapeutic effect in ameliorating diabetes-induced kidney damage. Furthermore, Msol and MS are identified as the primary anti-inflammatory active components of SWM-008 RMR.

## 5. Conclusions

In this investigation, STZ-NA induction was observed to significantly elevate the expression levels of IL-1β, IL-6, and COX-2 in the kidneys of rats, indicating an inflammatory response. However, dietary intervention with *M. pilosus* SWM-008-fermented RMR was found to effectively mitigate this upregulation of inflammatory markers. Particularly, the isolated compound Msol exhibited a notable reduction in the expression of renal inflammatory factors, including TNF-α, IL-1β, IL-6, and COX-2, showcasing its potent anti-inflammatory effects. Intriguingly, at equivalent dosages, Msol demonstrated a more substantial improvement in reducing IL-1β and COX-2 expressions compared to MS, highlighting its superior therapeutic efficacy. In histopathological tissue sections, the induced dose in this experiment unfortunately did not result in significant liver damage. However, the kidney tissue showed pathological signs, such as tubular swelling. Both Msol and MS also showed improvements in kidney pathology. These outcomes suggest that *M. pilosus* SWM-008-fermented RMR, through its functional components Msol and MS, effectively inhibits the expression of key inflammatory mediators, thereby providing a promising therapeutic strategy for alleviating diabetes-induced renal damage. The pronounced effects of Msol, in particular, suggest its potential as a novel functional pigment with significant implications in the treatment of diabetic nephropathy, paving the way for the development of new anti-inflammatory treatments based on this compound.

## Figures and Tables

**Table 1:** Effect of different dosage of *Monascus pilosus* SWM-008-fermented red mold rice, monascinol, and monascin on initial body weight, final body weight, liver weight, and kidney weight of STZ-NA-induced rats.

Groups	Initial Body Weight (g)	Final Body Weight (g)	Liver Weight (g)	Kidney Weight (g)
NOR	211.4 ± 5.8 ^a^	550.5 ± 35.1 ^b^	14.74 ± 1.86 ^b^	3.54 ± 0.51 ^a^
STZ-NA	208.3 ± 8.3 ^a^	288.3 ± 23.7 ^a^	11.57 ± 0.95 ^a^	3.51 ± 0.27 ^a^
RL	202.9 ± 10.6 ^a^	314.6 ± 31.8 ^a^	12.53 ± 1.06 ^a^	3.59 ± 0.34 ^a^
RH	202.5 ± 9.0 ^a^	311.5 ± 27.2 ^a^	12.60 ± 0.71 ^a^	3.59 ± 0.24 ^a^
Msol-1X	205.1 ± 7.9 ^a^	293.6 ± 30.4 ^a^	12.37 ± 1.42 ^a^	3.60 ± 0.23 ^a^
Msol-2X	202.5 ± 8.9 ^a^	297.3 ± 23.4 ^a^	12.31 ± 1.13 ^a^	3.48 ± 0.31 ^a^
MS-1X	201.8 ± 10.6 ^a^	286.1 ± 23.4 ^a^	12.10 ± 1.16 ^a^	3.38 ± 0.21 ^a^
MS-2X	200.5 ± 12.6 ^a^	293.1 ± 26.8 ^a^	12.20 ± 1.34 ^a^	3.38 ± 0.25 ^a^

Two groups of the rats daily administered with RO water (NOR group) or i.p. injected with 65 mg/kg b.w. STZ + 150 mg/kg b.w. NA to induce liver and kidney damage and daily administered with RO water (STZ-NA group). The other STZ-NA-induced rats were administered with a low dose (51.67 mg/kg b.w./day) (RL group) or high dose of *Monascus pilosus* SWM-008-fermented red mold rice (206.67 mg/kg b.w./day) (RH group), 1-fold dose monascinol (0.31 mg/kg b.w./day) (Msol-1X group), 2-fold dose monascinol (0.62 mg/kg b.w./day) (Msol-2X group), 1-fold dose monascin (0.31 mg/kg b.w./day) (MS-1X group), and 2-fold dose monascin (0.62 mg/kg b.w./day) (MS-2X group). Data are presented as means ± SD (n = 8). Mean values with different letters are significantly different (*p* < 0.05). The statistical significance in the biochemical effects was determined by ANOVA with Duncan’s multiple range test.

**Table 2:** Effect of different dosages of *Monascus pilosus* SWM-008-fermented red mold rice, monascinol, and monascin on food intake and water intake of STZ-NA-induced rats.

Groups	Food Intake (g)	Water Intake (mL)
NOR	1542.1 ± 126.6 ^a^	2953.8 ± 581.5 ^a^
STZ-NA	2350.1 ± 193.1 ^b^	10,821.3 ± 1295.8 ^b^
RL	2394.9 ± 200.6 ^b^	10,700.0 ± 1264.9 ^b^
RH	2464.5 ± 358.4 ^b^	11,330.0 ± 2220.8 ^b^
Msol-1X	2441.0 ± 279.7 ^b^	10,879.4 ± 1671.2 ^b^
Msol-2X	2424.5 ± 207.4 ^b^	11,190.0 ± 1314.4 ^b^
MS-1X	2359.8 ± 119.8 ^b^	10,264.0 ± 958.0 ^b^
MS-2X	2396.3 ± 275.0 ^b^	10,725.0 ± 1269.0 ^b^

Two groups of the rats daily administered with RO water (NOR group) or i.p. injected with 65 mg/kg b.w. STZ + 150 mg/kg b.w. NA to induce liver and kidney damage and daily administered with RO water (STZ-NA group). The other STZ-NA-induced rats were administered with a low dose (51.67 mg/kg b.w./day) (RL group) or high dose of *Monascus pilosus* SWM-008-fermented red mold rice (206.67 mg/kg b.w./day) (RH group), 1-fold dose monascinol (0.31 mg/kg b.w./day) (Msol-1X group), 2-fold dose monascinol (0.62 mg/kg b.w./day) (Msol-2X group), 1-fold dose monascin (0.31 mg/kg b.w./day) (MS-1X group), and 2-fold dose monascin (0.62 mg/kg b.w./day) (MS-2X group). Data are presented as means ± SD (n = 8). Mean values with different letters are significantly different (*p* < 0.05). The statistical significance in the biochemical effects was determined by ANOVA with Duncan’s multiple range test.

**Table 3:** Effect of different dosages of *Monascus pilosus* SWM-008-fermented red mold rice, monascinol, and monascin on serum AST, ALT, and albumin in the STZ-NA-induced rats.

Groups	AST (U/L)	ALT (U/L)	Albumin (g/dL)
NOR	104.3 ± 14.0 ^a^	37.9 ± 7.3 ^a^	5.03 ± 0.35 ^d^
STZ-NA	222.1 ± 19.4 ^d^	112.1 ± 17.5 ^c^	4.10 ± 0.32 ^a^
RL	169.8 ± 47.3 ^c^	85.8 ± 21.9 ^b^	4.20 ± 0.23 ^ab^
RH	146.3 ± 26.3 ^bc^	84.9 ± 12.0 ^b^	4.63 ± 0.14 ^c^
Msol-1X	154.4 ± 41.5 ^bc^	86.5 ± 20.9 ^b^	4.21 ± 0.10 ^ab^
Msol-2X	155.3 ± 30.4 ^bc^	89.9 ± 21.6 ^b^	4.31 ± 0.16 ^ab^
MS-1X	156.9 ± 26.0 ^bc^	86.5 ± 13.5 ^b^	4.44 ± 0.16 ^bc^
MS-2X	131.6 ± 39.5 ^ab^	91.3 ± 20.5 ^b^	4.41 ± 0.22 ^bc^

Two groups of the rats daily administered with RO water (NOR group) or i.p. injected with 65 mg/kg b.w. STZ + 150 mg/kg b.w. NA to induce liver and kidney damage and daily administered with RO water (STZ-NA group). The other STZ-NA-induced rats were administered with a low dose (51.67 mg/kg b.w./day) (RL group) or high dose of *Monascus pilosus* SWM-008-fermented red mold rice (206.67 mg/kg b.w./day) (RH group), 1-fold dose monascinol (0.31 mg/kg b.w./day) (Msol-1X group), 2-fold dose monascinol (0.62 mg/kg b.w./day) (Msol-2X group), 1-fold dose monascin (0.31 mg/kg b.w./day) (MS-1X group), and 2-fold dose monascin (0.62 mg/kg b.w./day) (MS-2X group). Data are presented as means ± SD (n = 8). Mean values with different letters are significantly different (*p* < 0.05). The statistical significance in the biochemical effects was determined by ANOVA with Duncan’s multiple range test.

**Table 4:** Effect of different dosages of *Monascus pilosus* SWM-008-fermented red mold rice, monascinol, and monascin on serum BUN, creatinine, and uric acid in the STZ-NA-induced rats.

Groups	BUN (mg/dL)	Creatinine (mg/dL)	Uric Acid (g/dL)
NOR	16.6 ± 3.2 ^a^	0.63 ± 0.08 ^d^	5.78 ± 0.96 ^c^
STZ-NA	45.5 ± 2.0 ^c^	0.56 ± 0.07 ^c^	3.23 ± 0.36 ^a^
RL	35.0 ± 6.1 ^b^	0.47 ± 0.06 ^b^	3.33 ± 0.31 ^a^
RH	37.2 ± 5.7 ^b^	0.48 ± 0.01 ^b^	3.48 ± 0.53 ^a^
Msol-1X	43.9 ± 9.3 ^c^	0.46 ± 0.04 ^ab^	4.41 ± 0.51 ^b^
Msol-2X	40.3 ± 5.9 ^bc^	0.49 ± 0.03 ^b^	4.51 ± 0.74 ^b^
MS-1X	36.5 ± 7.5 ^b^	0.48 ± 0.02 ^b^	4.49 ± 0.26 ^b^
MS-2X	36.5 ± 5.3 ^b^	0.42 ± 0.04 ^a^	4.41 ± 0.43 ^b^

Two groups of the rats daily administered with RO water (NOR group) or i.p. injected with 65 mg/kg b.w. STZ + 150 mg/kg b.w. NA to induce liver and kidney damage and daily administered with RO water (STZ-NA group). The other STZ-NA-induced rats were administered with a low dose (51.67 mg/kg b.w./day) (RL group) or high dose of *Monascus pilosus* SWM-008-fermented red mold rice (206.67 mg/kg b.w./day) (RH group), 1-fold dose monascinol (0.31 mg/kg b.w./day) (Msol-1X group), 2-fold dose monascinol (0.62 mg/kg b.w./day) (Msol-2X group), 1-fold dose monascin (0.31 mg/kg b.w./day) (MS-1X group), and 2-fold dose monascin (0.62 mg/kg b.w./day) (MS-2X group). Data are presented as means ± SD (n = 8). Mean values with different letters are significantly different (*p* < 0.05). The statistical significance in the biochemical effects was determined by ANOVA with Duncan’s multiple range test.

## Data Availability

All data included in this study are available upon request by contacting the corresponding author.

## Abbreviations

Advanced glycation end products (AGEs), alanine transaminase (ALT), ankaflavin (AK), aspartate aminotransferase (AST), blood urea nitrogen (BUN), cyclooxygenase-2 (COX-2), deferricoprogen (DFC), diabetic kidney disease (DKD), diabetic liver disease (DLD), diabetic nephropathy (DN), dimerumic acid (DMA), enzyme-linked immunosorbent assay (ELISA), enzymes glutathione (GSH), glutathione reductase (GRd), hematoxylin and eosin staining (H&E staining), heme oxygenase-1 (HO-1), hepatic glucose production (HGP), interleukin-1β (IL-1β), interleukin-6 (IL-6), monacolin K (MK), monascin (MS), monascinol (Msol), nicotinamide (NA), reactive oxygen species (ROS), red mold rice (RMR), streptozotocin (STZ), tumor necrosis factor-α (TNF-α), γ-amino butyric acid (GABA).
